# Protein S-acyltransferases and acyl protein thioesterases, regulation executors of protein S-acylation in plants

**DOI:** 10.3389/fpls.2022.956231

**Published:** 2022-07-27

**Authors:** Jincheng Li, Manqi Zhang, Lijuan Zhou

**Affiliations:** College of Forestry, Co-Innovation Center for the Sustainable Forestry in Southern China, Nanjing Forestry University, Nanjing, China

**Keywords:** protein S-acyltransferases, acyl protein thioesterases, protein S-acylation, plant, signal transduction, post-translational modification

## Abstract

Protein S-acylation, also known as palmitoylation, is an important lipid post-translational modification of proteins in eukaryotes. S-acylation plays critical roles in a variety of protein functions involved in plant development and responses to abiotic and biotic stresses. The status of S-acylation on proteins is dynamic and reversible, which is catalyzed by protein S-acyltransferases (PATs) and reversed by acyl protein thioesterases. The cycle of S-acylation and de-S-acylation provides a molecular mechanism for membrane-associated proteins to undergo cycling and trafficking between different cell compartments and thus works as a switch to initiate or terminate particular signaling transductions on the membrane surface. In plants, thousands of proteins have been identified to be S-acylated through proteomics. Many S-acylated proteins and quite a few PAT-substrate pairs have been functionally characterized. A recently characterized acyl protein thioesterases family, ABAPT family proteins in *Arabidopsis*, has provided new insights into the de-S-acylation process. However, our understanding of the regulatory mechanisms controlling the S-acylation and de-S-acylation process is surprisingly incomplete. In this review, we discuss how protein S-acylation level is regulated with the focus on catalyzing enzymes in plants. We also propose the challenges and potential developments for the understanding of the regulatory mechanisms controlling protein S-acylation in plants.

## Introduction

Protein S-acylation, referring to the addition of 16-carbon palmitate (predominant form) or 18-carbon stearate to specific cysteine residues *via* a thioester bond, has attracted much attention due to its exclusively reversible feature among lipid modifications and its vital roles in a variety of biological processes (Smotrys and Linder, 2004; Running, 2014; Li and Qi, 2017; Zheng et al., 2019; Hu and Ye, 2022). The status of S-acylation on proteins is dynamically changed, which is catalyzed by protein acyltransferases (PATs) and de-S-acylation enzymes, such as acyl-protein thioesterases (APTs) and recently reported α/α Hydrolase domain-containing Protein 17-like acyl protein thioesterases (ABAPTs; Liu et al., 2021). In plants, owing to the application of approaches for studying S-acylation and remarkable advances in high-resolution proteomics, enormous progress has been made in understanding S-acylation in the last two decades. Thousands of proteins have been identified as potential S-acylated proteins involved in vesicle trafficking, signal transduction, primary and secondary metabolism, and stress responses (Hemsley et al., 2013; Srivastava et al., 2016; Zhou et al., 2020; Kumar et al., 2022). S-acylated proteins and their functions have been well studied and nicely reviewed elsewhere (Hemsley et al., 2008; Li and Qi, 2017; Turnbull and Hemsley, 2017; Zheng et al., 2019). In this paper, we will review the current progress of S-acylation studies from the standpoint of the regulatory mechanisms of the protein S-acylation and de-S-acylation process with a focus on their catalyzing enzymes in plants.

### Protein sequence requirements for S-acylation

S-acylation is a widespread modification found in a variety of protein families. Functionally characterized S-acylated proteins are summarized in Table 1, including heterotrimeric G proteins, small G proteins Rho of Plants (ROPs), Calcineurin B-like proteins (CBLs), calcium-dependent protein kinases (CPKs), receptor-like kinases (RLKs), receptor-like cytoplasmic kinases (RLCKs), remorins, cellulose synthase subunits (CESAs), transcription factors (TFs), protein phosphatase type 2C proteins (PP2C), etc. Among these proteins, the S-acylation sites have been validated and functionally studied by point mutagenesis. In addition to these listed S-acylation sites, there may exist other uncharacterized modified sites in these proteins. Except for the requisite cysteines, no well-defined conserved motifs have been found for this modification so far. Nevertheless, certain motifs and some other post-translational modifications (PTMs) are required for the S-acylation of particular proteins.

**Table 1:** Functionally characterized S-acylated proteins in plants.

Gene family	Proteins	Organism	S-acylation sites	Dual lipidation	References
Hetero-trimeric G protein	GPA1	*Arabidopsis*	N-terminal C5	myristoyl	Adjobo-Hermans et al. (2006)
	AGG2	*Arabidopsis*	C95	prenyl	Zeng et al. (2007) and Hemsley et al. (2008)
ROP GTPase	ROP2	*Arabidopsis*	G domain C20, 157	prenyl	Chai et al. (2016)
	ROP6	*Arabidopsis*	G domain C21, 156	prenyl	Sorek et al. (2010, 2011a,b, 2017)
	ROP9/RAC7	*Arabidopsis*	C-terminal C196, 203, 206		Lavy et al. (2002) and Lavy and Yalovsky (2006)
	ROP10/RAC8	*Arabidopsis*	C-terminal C199, 205		Lavy et al. (2002) and Lavy and Yalovsky (2006)
	ROP11/RAC10	*Arabidopsis*	C-terminal C202, 208		Lavy et al. (2002)
	ZmROP6	Maize	C-terminal C199, 206, 210		Ivanchenko et al. (2000)
	ZmROP7	Maize	C-terminal C199, 206, 210		Ivanchenko et al. (2000)
Rab GTPase	Ara6	*Arabidopsis*	N-terminal C3	myristoyl	Ueda et al. (2001)
CBLs	CBL1	*Arabidopsis*	N-terminal C3	myristoyl	Batistic et al. (2008)
	CBL2	*Arabidopsis*	N-terminal C4, 12, 18		Batistič et al. (2012) and Zhou et al. (2013)
	CBL3	*Arabidopsis*			Zhou et al. (2013)
	CBL4/SOS3	*Arabidopsis*	N-terminal C3	myristoyl	Batistic et al. (2008) and Villalta et al. (2021)
	CBL5	*Arabidopsis*	N-terminal C3, 5	myristoyl	Batistic et al. (2008) and Saito et al. (2018)
	CBL6	*Arabidopsis*	N-terminal C5, 19, 20		Zhang et al. (2017) and Zhou et al. (2013)
	CBL9	*Arabidopsis*	N-terminal C3	myristoyl	Batistic et al. (2008)
	CBL10	*Arabidopsis*	N-terminal C38		Chai et al. (2020)
	OsCBL2	Rice	N-terminal C4, 18, 19		Tian et al. (2022)
	OsCBL3	Rice	N-terminal C4, 12, 18, 19		Tian et al. (2022)
	MdCBL1	Apple	N-terminal C3		Jiang et al. (2021)
CPKs	CPK6	*Arabidopsis*	N-terminal C5	myristoyl	Saito et al. (2018)
	OsCPK2	Rice	N-terminal C3, 4	myristoyl	Martín and Busconi (2000)
	ZmCPK9	Maize	N-terminal C3, 4		Zhang et al. (2020)
	LeCPK1	Tomato	N-terminal C4, 5	myristoyl	Leclercq et al. (2005)
	MtCPK3	Medicago	N-terminal C3	myristoyl	Gargantini et al. (2006)
RLK	FLS2	*Arabidopsis*	C830, C831		Hurst et al. (2019)
	P2K1	*Arabidopsis*	C394, C407		Chen et al. (2021)
RLCKs	PBS1	*Arabidopsis*	N-terminal C3, 6		Qi et al. (2014) and Liu et al. (2021)
	PBL1	*Arabidopsis*	N-terminal C4	myristoyl	Ranf et al. (2014) and Liu et al. (2021)
	BIK1	*Arabidopsis*	N-terminal C4	myristoyl	Ranf et al. (2014) and Liu et al. (2021)
	HIR2	*Arabidopsis*	N-terminal C6, 7		Liu et al. (2021)
	STRK1	*Arabidopsis*	N-terminal C5, 10, 14		Zhou et al. (2018)
	LIP1	*Arabidopsis*	N-terminal C7, 10		Liu et al. (2013)
	LIP2	*Arabidopsis*	N-terminal C3, 6		Liu et al. (2013)
	SGN1	*Arabidopsis*	N-terminal C23, 24		Alassimone et al. (2016)
Remorins	MtSYMREM	Medicago	C-terminal C197		Konrad et al. (2014)
	NbREM1	Tobacco	C-terminal C206		Fu et al. (2018)
	OsREM1.4	Rice	C-terminal C200		Fu et al. (2018)
	OsGSD1	Rice	C-terminal C524, 527		Gui et al. (2015)
CESAs	CESA7	*Arabidopsis*	VR2 region C621, 623, 624, 626; CT region C1022, 1,026		Kumar et al. (2016, 2022)
	CESA4	*Arabidopsis*	RING finger region		Kumar et al. (2022)
	CESA8	*Arabidopsis*	RING finger region		Kumar et al. (2022)
endoglucanase	KOR1	*Arabidopsis*	C64		Kumar et al. (2022)
nuclease	CAN1	*Arabidopsis*		myristoyl	Leśniewicz et al. (2012)
	CAN2	*Arabidopsis*		myristoyl	Leśniewicz et al. (2012)
TFs	MfNACsa	Medicago	N-terminal C26		Duan et al. (2017)
	OsNAC9	Rice			Tian et al. (2022)
PP2C	POL	*Arabidopsis*	N-terminal C11	myristoyl	Gagne and Clark (2010)
	PLL1	*Arabidopsis*	N-terminal C11	myristoyl	Gagne and Clark (2010)
Phosphor-lipase C	NPC4	*Arabidopsis*	C-terminal C533		Yang et al. (2021)
	BnaC01.NPC4	*Brassica*	C-terminal C531		Yang et al. (2021)
Nitric oxide synthase	NOA1	*Arabidopsis*	C107 C108		Lai et al. (2015)	OsNOA1	Rice			Tian et al. (2022)
	Heavy metal-associated isoprenylated plant protein	NbHIPP26	tobacco	N terminal C13		Cowan et al. (2018)
R protein	RIN4	*Arabidopsis*	C-terminal C203, 204, 205		Kim et al. (2005) and Liu et al. (2021)
NBS-LRR	GmRsc4-3	Soybean	N terminal C28 C29		Yin et al. (2021)
Pathogen effectors	AvrPphB	*Pseudomonas*	C64	myristoyl	Dowen et al. (2009)
	ORF4	*Pseudomonas*	C113	myristoyl	Dowen et al. (2009)
	NopT	*Sinorhizobium*	C51, C52	myristoyl	Dowen et al. (2009)
	BSCTV C4	*Beet severe curly top virus*	N-terminal C8	myristoyl	Li et al. (2018)
	EACMCV AC4	*East African cassava mosaic Cameroon virus*	N-terminal C3	myristoyl	Fondong et al. (2007)
	MYMV AC4	*Mungbean yellow mosaic virus*	C11		Carluccio et al. (2018)

Other lipid modification, either N-myristoylation or prenylation, frequently occurs together with S-acylation, and even acts as a prerequisite for S-acylation. In most reports, S-acylation itself or in combination with other lipidations are required for the correct trafficking and membrane targeting of the modified proteins; mutations at either the S-acylation site, the other lipidation site, or the dual lipidation sites show incorrect subcellular localization and compromised functions of the protein (Adjobo-Hermans et al., 2006; Batistic et al., 2008; Li and Qi, 2017; Hemsley, 2020).

Unlike S-acylation, N-myristoylation and prenylation are catalyzed by their enzymes on relatively conserved sequences. N-myristoylation refers to the addition of 14-carbon myristoyl groups to N-terminal glycine residues of proteins that harbor an MGXXXS/T motif. In contrast, prenylation modifies target proteins in the C-terminus by adding a 15-carbon farnesyl or a 20-carbon geranylgeranyl group to the cysteine of the C-terminal ‘CaaX’ box (Sorek et al., 2009; Running, 2014; Hemsley, 2015). As shown in Table 1, normally, S-acylation sites occur proximal to the N-myristoylation site when dual lipidation occurs, which is not applicable to the prenylation-S-acylation combination. Proteins from a gene family/subfamily are likely subjected to the same dual lipidation types due to the sequence and functional conservations (Table 2).

**Table 2:** Functionally characterized PATs in plants.

Organism	Gene	AGI locus	Subcellular localization	Substrates	Auto-S-acylation sites	Involved biological processes	References
*Arabidopsis*	PAT4	At3g56930	PM, Golgi, early endosomes	ROP2		Root hair growth	Batistič et al. (2012) and Wan et al. (2017a,b)
	PAT5	At3g48760	PM	P2K1	C188	Immune responses	Batistič et al. (2012) and Chen et al. (2021)
	PAT9	At5g50020	PM	P2K1	C166	Immune responses	Batistič et al. (2012) and Chen et al. (2021)
	PAT10	At3g51390	tonoplast, Golgi	CBL2, CBL3, CBL6, CBL10	C192	Plant development and salt stress	Batistič et al. (2012), Qi et al. (2013), Zhou et al. (2013), and Chai et al. (2020)
	PAT13	At4g22750	PM and vesicles	NOA1		Leaf senescence	Batistič et al. (2012) and Lai et al. (2015)
	PAT14	At3g60800	Golgi, prevacuolar compartments	NOA1	C157	Leaf senescence	Batistič et al. (2012), Lai et al. (2015), Li et al. (2016), and Zhao et al. (2016)
	PAT15	At5g04270	ER and Golgi apparatus		C122	Lipid catabolism during early seedling growth	Batistič et al. (2012) and Li et al. (2019a)
	PAT21	At2g33640	PM		C174	Sexual reproduction	Batistič et al. (2012) and Li et al. (2019b)
	PAT24/TIP1	At5g20350	Golgi, non-Golgi vesicles			Root hair growth	Batistič et al. (2012) and Hemsley et al. (2005)
Rice	OsPAT15	Os02g0819100				Plant architecture	Peng et al. (2018) and Zhou et al. (2017)
	OsDHHC13	Os04g0674450	PM	OsNAC9			Yuan et al. (2013) and Tian et al. (2022)
	OsDHHC14	Os05g0436900	PM	GSD1			Yuan et al. (2013) and Tian et al. (2022)
	OsDHHC18	Os07g0467800	endomembrane	OsNOA1			Yuan et al. (2013) and Tian et al. (2022)
	OsDHHC30	Os12g0480000	endomembrane	OsCBL2, OsCBL3	C182	Salt and oxidative tolerance	Yuan et al. (2013) and Tian et al. (2022)
Maize	ZmTIP1	Zm00001d046590	PM, prevacuolar compartments, Golgi	ZmCPK9		Root hair elongation and drought tolerance	Yuan et al. (2013) and Zhang et al. (2020)
	ZmPAT14	Zm00018ab089390				Leaf senescence	Zhao et al. (2016)
Wheat	TaPAT14					Leaf senescence	Zhao et al. (2016)
Pear	PbPAT14	Pbr041901.1				ABA pathway	Pang et al. (2019)
Apple	MdPAT16	Md10g1058600	PM	MdCBL1	C244	Salt stress, accumulation of sugars	Jiang et al. (2021)

N-myristoylation-S-acylation combination has been found in the Gα subunit GPA1 in the heterotrimeric G proteins, Rab GTPase ARA6, CBLs, CPKs, RLCKs, staphylococcal-like nucleases, PP2Cs, and some of the pathogen effectors (Ueda et al., 2001; Adjobo-Hermans et al., 2006; Batistic et al., 2008; Dowen et al., 2009; Gagne and Clark, 2010; Leśniewicz et al., 2012; Liu et al., 2013; Saito et al., 2018). The S-acylation sites are always located adjacent to or nearby the N-myristoylated glycine (G2 site) at the cysteine residues from C3 to C11. This rule also applies to the bacterial type III effectors, including AvrPphB, ORF4, and NopT, after undergoing auto-proteolysis on specific sites (Dowen et al., 2009). N-myristoylation and S-acylation of calcium signaling participants, CBLs and CPKs, have been well studied. CBL1, 4, 5, and 9, which undergo both N-myristoylation and S-acylation, are localized on the plasma membrane (PM), whereas CBL2, 3, 6, and 10, containing only S-acylation modification at the N-terminal sites, are found on the tonoplast (Zhou et al., 2013; Saito et al., 2018; Chai et al., 2020; Villalta et al., 2021). Mutations at any of these sites significantly diminish the protein localization. It seems that S-acylation sites directly affect the membrane localization of CBLs, and myristoylation appears to direct their targeting to the PM or tonoplast. The case of CBLs is consistent with the prevailing model that the major function of myristoylation is to assist in the targeting of the modified protein to the membrane, and the addition of S-acylation can serve to stabilize the membrane association of a myristoylated protein (Farazi et al., 2001; Resh, 2013). However, many exceptions exist. PBS1 contains predicted N-terminal myristoylation and S-acylation signal. However, the myristoylation of PBS1 was not detected experimentally and mutation of the G2 site does not affect the protein localization on PM, indicating that the N-terminal S-acylation of itself is sufficient for PM localization of PBS1, which is independent of myristoylation (Dowen et al., 2009; Qi et al., 2014). Rab GTPase protein Ara6 is modified by both N-myristoylation and S-acylation. The Ara6^G2A^ mutant was not S-acylated, indicating that myristoylation of Ara6 is a prerequisite for S-acylation (Ueda et al., 2001). Pathogens employ the host lipidation machinery to modify the effectors and thus anchor them on plant cellular PM for further avirulence activities. It has been demonstrated that the self-proteolysis process next to the GDK motif of AvrPphB, the VER motif of ORF4, and the DKM motif of NopT, exposes their respective embedded sites for fatty acylation. Therefore, these motifs and the auto-proteolysis are a prerequisite for dual lipidations and the acylation-dependent PM localization (Dowen et al., 2009).

Prenylation-S-acylation combination has been well characterized in Gγ subunit AGG2 in the heterotrimeric G proteins and type-I ROPs. S-acylation sites of ROPs are uniformly located at the protein C-terminus, but the two subfamilies undergo different mechanisms of membrane targeting. Type-I ROPs, including ROP1-ROP8, which terminate with a conserved CaaL box and a polybasic region, undergo prenylation and S-acylation on the G domain (Sorek et al., 2010, 2011a,b, 2017). PM localization of Type-II ROPs, including ROP9, ROP10, and ROP11, requires S-acylation on two or three C-terminal cysteines on the GC-CG box and is prenylation independent, even though ROP9 contains a CaaX box and could be prenylated in yeast (Lavy et al., 2002). Interestingly, heterotrimeric G-protein subunits undergo different lipidations. Gα subunit GPA1 is both myristoylated and S-acylated (Adjobo-Hermans et al., 2006); Gγ subunit AGG1 is prenylated while AGG2 is simultaneously prenylated and S-acylated. Moreover, polybasic residues also contribute to the efficient membrane-targeting of AGG2 (Zeng et al., 2007).

Dual lipidation or multiple adjacent S-acylations may enhance membrane attachment or trafficking of proteins by creating a more hydrophobic protein structure. Consistent with this assumption, consecutive or adjacent modification of S-acylation are commonly found in S-acylated proteins, especially for soluble proteins, e.g., double/triple/quad S-acylation sites in ROPs, CBLs, some RLCKs, CESAs, and triple S-acylation sites in the C-terminal domain of RIN4 (Lavy et al., 2002; Kim et al., 2005; Zhou et al., 2018; Alam et al., 2021; Tian et al., 2022).

Based on the functionally characterized sites, unlike myristoylation and prenylation, S-acylation modification is not restricted to the protein ends. The proteomics data strongly support it. A most recent acyl-RAC-based site-specific S-acylproteome analysis for *Arabidopsis* identified 1849 putative S-acylated cysteine sites from 1,094 proteins with high- and medium-confidence, representing around 6% of the detectable *Arabidopsis* proteome. These putative S-acylated proteins have diverse molecular functions and are from almost all cellular compartments including soluble and transmembrane proteins (Kumar et al., 2022). Cellulose synthase A proteins, including CESA4, CESA7, and CESA8, are S-acylated in both the centrally located variable region 2 (VR2) and the carboxy-terminal domain (Kumar et al., 2016, 2022). Several S-acylation sites have been found in both the kinase domain and non-kinase domains of BSK proteins, which belong to the RLCK family (Ren et al., 2019; Kumar et al., 2022). For RLKs, S-acylation occurs in the kinase domain, at the juxta-membrane sites in the intracellular domain, or in the extracellular domain (Kumar et al., 2022).

### Protein S-acyltransferases and their substrates in plants

Protein S-acyltransferase (PAT) family proteins, featured by a highly conserved Asp-His-His-Cys cysteine-rich domain (DHHC-CRD), are responsible for protein S-acylation. Plant PATs are transmembrane proteins with 3–6 membrane-spanning domains and possess variable N-and C-terminal domains that might be essential for their substrate specificity (Smotrys and Linder, 2004; Batistič et al., 2012). Functions of PAT proteins from different organisms are highly conserved as evidenced by the complemented assay for the yeast akr mutant. Based on studies from the mammalian and yeast systems, the PATs catalyze target proteins through a two-step ping-pong mechanism: (1) the deprotonated and nucleophilic cysteine in the catalytic central DHHC tetrapeptide, undergoes auto-acylation and (2) transfer the acyl group to a cysteine residue on a target protein (Jennings and Linder, 2012; Zmuda and Chamberlain, 2020). Mutation analyses revealed that the cysteine residue of the DHHC domain is necessary for PAT auto-acylation and its enzyme activity (Peng et al., 2018; Li et al., 2019b; Chen et al., 2021; Jiang et al., 2021; Tian et al., 2022).

#### PATs in *Arabidopsis*

In *Arabidopsis*, the PAT family comprises 24 members (Batistic, 2012). Microarray data suggests that most of the *Arabidopsis* PATs display a broad and constant expression pattern at different developmental stages. Only a few PAT genes exhibited a specific transcription either in certain tissues and/or at a specific developmental stage. PATs are localized to a variety of intracellular membranes, including PM, ER, Golgi, tonoplast, and vesicles, indicating that proteins could possibly be S-acylated during the protein PTM and maturation processes on ER and Golgi, or during protein sorting and trafficking processes in Golgi and vesicles, or after arriving targeting compartments, such as PM and tonoplast (Batistič et al., 2012). Based on the subcellular localization, protein PTM time point, and the necessity for a functional protein, we propose that PATs localized on ER (PAT3, 15, 17, and 18), Golgi (PAT14, 15, 16, 23, and 24), and vesicles (PAT1, 2) may undertake most constant S-acylation modification that are necessary for protein maturation, sorting, and targeting; PATs localized on PM (PAT4, 5, 6, 7, 8, 9, 13, 19, 20, 21, and 22) and tonoplast (PAT10, 11, 12) may be responsible for local routine S-acylation processes and/or for protein S-acylation in response to external stimuli. Interestingly, about half of plant PATs reside on PM, indicating that PM might be one of the main locations for S-acylation occurrence and also suggesting the importance of S-acylation for the functions of PM proteins in plants. Since the number of S-acylated proteins far exceeds the number of PATs, PATs are likely to S-acylate multiple substrates (Hemsley, 2020). PATs may act redundantly due to their overlapping substrates.

Several PAT mutants from different plant species have been reported to show visible phenotypes and their substrates have been characterized. TIP1/PAT24 is the first reported PAT in plants, whose mutant *tip1* shows growth defects including dwarf plants, smaller rosettes, and shorter root hair (Hemsley et al., 2005). TIP1 plays a key role in root hair growth polarity since *tip1* mutants exhibited root hairs with multiple initiations and with branches (Hemsley et al., 2005; Zhang et al., 2015). Interestingly, TIP1 acts synergistically with PLURIPETALA (PLP), which involves in protein prenylation, during root hair growth (Chai et al., 2016). Besides TIP1, PAT4 also regulates root hair growth. PAT4 S-acylates ROP2 and facilitates the PM association of ROP2 at the root hair apex and thus regulates root hair growth (Wan et al., 2017a). In addition, PAT4 also functions in apex-associated re-positioning of the nucleus during root hair elongation (Wan et al., 2017b). PAT10 is critical for plant development and salt tolerance in *Arabidopsis* by regulating the tonoplast localization of several CBLs, including CBL2, CBL3, CBL6, and CBL10, whose membrane association depends on S-acylation (Qi et al., 2013; Zhou et al., 2013; Chai et al., 2020). PAT13 and PAT14 are involved in leaf senescence regulation by S-acylating chloroplast-localized Nitric Oxide Associated 1 (NOA1). *pat13/pat14* double mutant displays a severely early leaf senescence phenotype (Lai et al., 2015). PAT13 is localized on PM and vesicles, PAT14 is localized on Golgi and vesicles, while NOA1 functions on chloroplast. It has been proposed that NOA1 is S-acylated by PAT13 and PAT14 during trafficking to chloroplasts by the endomembrane system (Lai et al., 2015). Interestingly, Li et al. (2016) and Zhao et al. (2016) discovered a different regulatory mechanism of PAT14 in leaf senescence regulation. They found that *pat14* mutants were hypersensitive to salicylic acid (SA) and SA pathway-related genes were altered in *pat14* mutants. Repressing SA biosynthesis or signaling by knock-out SA receptor *NPR1* or SA hydroxylase *NahG* in *pat14* can rescue its early senescence phenotype, demonstrating that AtPAT14 regulates senescence *via* SA pathways (Li et al., 2016; Zhao et al., 2016). In addition, *PAT14* homologs from maize (*ZmPAT14*) and wheat (*TaPAT14*) fully rescued the precocious leaf senescence of *Arabidopsis pat14*, indicating the conservative role of PAT14 in regulating leaf senescence between dicots and monocots (Zhao et al., 2016). PAT5 and PAT9 are two of the few well-studied PATs in *Arabidopsis* (Chen et al., 2021). These two enzymes were shown to S-acylate the extracellular ATP receptor P2K1 at the PM to regulate the pathogen immune response in *Arabidopsis*. Interaction between PAT5, 9, and P2K1 is intricate and fine-tuned. PAT5 and PAT9 were activated by P2K1 phosphorylation, and activated PAT5, 9, in turn, impacted S-acylation level of P2K1. S-acylation of P2K1 plays an important role in mediating ATP-induced autophosphorylation and protein turnover, thus affecting the responses to extracellular ATP and pathogen infection (Chen et al., 2021). This study firstly reported the regulation of PAT activity by phosphorylation. Besides, functions of PAT15 and PAT21 have been studied, but their substrates have yet to be uncovered. PAT15 is involved in seed triacylglycerol catabolism during early seedling growth (Li et al., 2019a). AtPAT21 is essential for both male and female gametogenesis by participating in repairing SPO11-induced meiotic DNA double-stranded breaks (Li et al., 2019b).

#### PATs in other higher plants

In addition to *Arabidopsis*, the *PAT* gene family has also been found in other higher plants (Yuan et al., 2013). There are 30 PATs in rice (*Oryza sativa* L.). A quick screening of PATs of proven S-acylated proteins in rice through constructing an *OsDHHC* cDNA library and the BiFC assay has identified several PAT-substrate pairs OsDHHC13-OsNAC9, OsDHHC14-OsGSD1, OsDHHC18-OsNOA1, and OsDHHC30-OsCBL2, 3 (Tian et al., 2022). Further functional characterization of OsDHHC30-OsCBL2, 3 pairs has proven that OsDHHC30 determines the endomembrane association of OsCBL2 and OsCBL3 by S-acylation and contributes to salt tolerance in rice (Tian et al., 2022). OsPAT15, an alternatively spliced model of Os02g0819100 in rice, is a homolog of AtPAT15/AtDHHC1 and contains a zinc finger DHHC domain. OsPAT15 regulates plant architecture by altering the rice tiller (Zhou et al., 2017). Heterologous overexpression of the *OsPAT15* gene into the dicot *Brassica napus* L. exhibited increased primary branches and increased seed yield (Peng et al., 2018). In *B. napus* L., 24 proteins have been predicted as PATs based on protein sequences. Except for BnPAT3, 8, 18, 19, 20, 21, and 22, all the other BnPATs contain the DHHC-CRD domain. No function studies have been done for these genes (Yuan et al., 2013; Peng et al., 2018). ZmTIP1, a homolog of AtTIP1/PAT24, was reported to positively regulate root hair length and drought tolerance in maize (*Zea mays* L.) through S-acylating its substrate ZmCPK9, which facilitates the association of ZmCPK9 with the PM (Zhang et al., 2020). MdPAT16, a PAT member in apple (*Malus domestica*), was shown to regulate plant resistance to salt stress and the accumulation of soluble sugars by S-acylating MdCBL1 protein. S-acylation of MdCBL1 at the N-terminal site enhances the protein stability and the PM localization (Jiang et al., 2021). *PbPAT14*, an S-acyltransferase gene from pear (*Pyrus bretschneideri*), has been functionally characterized. Knockout transgenic pear lines of *PbPAT14* using CRISPR/Cas9 system exhibited yellow dwarf phenotype. Endogenous hormone measurement and marker gene expression analysis indicated that PbPAT14 function was related to the ABA pathway (Pang et al., 2019).

### de-S-acylation enzymes and their substrates in plants

**Figure 1:**
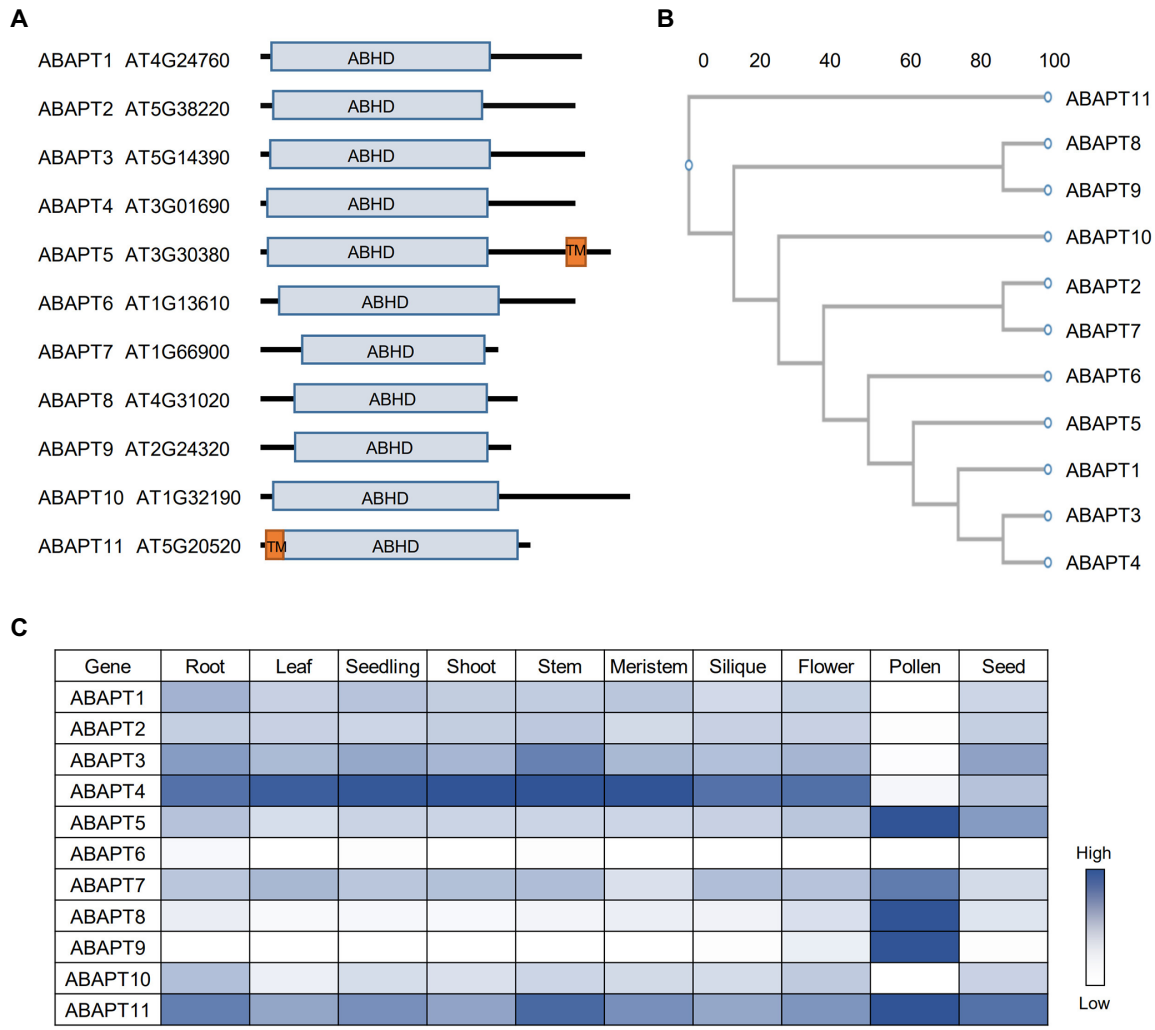
The ABAPT family in *Arabidopsis*. **(A)** Protein domain architecture of ABAPT family proteins. ABHD, alpha beta hydrolase domain; TM, transmembrane domain. **(B)** Phylogenetic tree of the *Arabidopsis* ABAPT family proteins. The phylogenic tree was conducted using protein sequences and the neighbor-joining method was used. Scale bar indicates genetic distance. **(C)** Expression profiles of all ABAPT genes in different tissues. The heat map shows the relative expression level of each gene. All the gene expression data were retrieved from *Arabidopsis* RNA-seq Database (http://ipf.sustech.edu.cn/pub/athrna/).

Protein de-S-acylation is the other half indispensable process in the S-acylation/de-S-acylation cycle, which modulates protein membrane localization and protein properties by removing the thioester-linked fatty acids from protein substrates (Figure 1). In mammals, the G proteins, Ras-family small GTPases, and N-Ras have established a distinct mode of membrane protein directionality depending on an S-acylation and de-S-acylation cycle (Duncan and Gilman, 1998; Won et al., 2018). The Protein de-S-acylation seems to occur everywhere in the cell, and no specific conserved sequence or biunique substrate specificity has been described for this enzymatic reaction so far, which is also reflected by the fact that thousands of proteins could be S-acylated, but only a few acyl protein thioesterases have been identified.

Compared to a significant number of studies in mammals, the de-S-acylated proteins and related enzymes have been rarely reported in plant cells. Unlike the plant PAT family genes, which are highly homologous to PATs from mammals and yeast, there are no homologs in plants with high similarity to mammalian APT1/2 (Hemsley, 2020). Nevertheless, MtAPT1, a putative homolog of human APT1 in *Medicago falcata*, is the first reported candidate acyl protein thioesterases in plant. MtAPT1 mediates the nuclear translocation of a NAC transcription factor MfNACsa in response to drought stress, which is suggested to be realized by its de-S-acylation (Duan et al., 2017). However, unlike mammalian APTs, MtAPT1 belongs to an acyl-ACP thioesterase from the Hotdog fold superfamily rather than the serine hydrolase superfamily. Further biochemical evidence is needed to support the role of MtAPT1 in de-S-acylation (Duan et al., 2017; Hemsley, 2020). Bürger et al. suggested ZmB6T1C9 be a *bona fide* APT member based on the phylogenetic and structural analysis of plant hydrolases. Due to the structural similarity to human APT2, ZmB6T1C9 was also assigned as ZmAPT2 (Bürger et al., 2017). However, the de-S-acylation activity, substrates, and biological functions of this enzyme have not been studied.

Recently, ABAPT family proteins (ABHD17-like acyl protein thioesterases, ABAPTs), which share a conserved ABHD region with mammalian ABHD17 proteins, were identified as putative protein acyl thioesterases in *Arabidopsis* (Liu et al., 2021). There are 11 members in the ABAPT family (Figure 1A), and all of them share a conserved ABHD protein domain with a serine, an aspartate, and a histidine residue, which are proved to be essential for ABDH17 de-S-acylation activity (Lin and Conibear, 2015). The de-S-acylation activity of ABAPTs was tested and the ABAPT-substrate pairs were identified using a robust screen system, in which five RFP-tagged plant immunity-related protein substrate candidates (RIN4, PBS1, PBL1, HIR2, and BIK1) were co-expressed with ABAPTs in protoplasts and the effects of ABAPTs on the subcellular localization and S-acylation level of these candidate substrates were examined. By this method, these S-acylated proteins were proved to undergo a de-S-acylation process mediated by ABAPTs (Liu et al., 2021). In addition, ABAPT8-RIN4/PBL1/BIK1, ABAPT7-HIR2, and ABAPT11-PBS1 catalytic pairs have been identified. A further functional study demonstrated that de-S-acylation of RIN4 mediated by ABAPT8 stimulated cell death, implying that ABAPT8 may function in the regulation of plant immunity responses (Liu et al., 2021). Although the ABHD region of ABAPTs is conserved with mammalian ABHD17 proteins, the other regions show very low levels of identity. The striking differences between ABAPTs and mammalian ABHD17 proteins suggest distinct mechanisms of the thioesterase activity regulation or/and substrate selectivity.

Among the ABAPT family, ABAPT5 and ABAPT11 harbor a transmembrane domain, while other ABAPTs do not (Figure 1A). Interestingly, all 11 ABAPTs could localize on the plasma membrane, which is probably implemented by their own S-acylation modification similar to their mammalian homologs. However, future biochemical evidence is needed to prove their S-acylation. Other than the membrane localization, the ABAPTs are also localized in the cytosol (Liu et al., 2021), reflecting the diverse subcellular localization of the detected S-acylated proteins (Hemsley et al., 2013; Kumar et al., 2022). In mammals, APT1 is even an active mitochondrial acyl thioesterases (Kathayat et al., 2018). It would be interesting to investigate the roles of ABAPTs in distinct cell organelles other than plasma membrane.

The spatial and temporal expression patterns of genes are very important for their cellular functions. In *Arabidopsis*, similar to PATs, the ABAPT expression could also be detected in different *Arabidopsis* tissues and developmental stages (Figure 1C). ABAPTs can be divided into three groups based on their expression patterns: (1) ABAPT5/7/8/9/11 are predominantly expressed in pollen; (2) ABAPT1/2/3/4/10 are constantly expressed in other tissues except pollen; and (3) ABAPT6 shows fairly low or undetectable expression in all tissues shown here. ABAPT11 was previously identified as Wavy Growth 2 (WAV2), which functions in root bending in response to environmental stimuli (Mochizuki et al., 2005). However, the function of WAV2 in pollen has not been studied. Interestingly, ABAPT5 and ABAPT11, the two putative transmembrane domain-containing proteins, are highly expressed in pollen. ABAPT1, ABAPT3, and ABAPT4 are clustered into one sub-branch in the phylogenetic tree (Figure 1B), and they display similar expression patterns, suggesting that they may act redundantly in plants. ABAPT8 and ABAPT89 are located in a sub-branch and also share similar expression patterns, which may suggest functional redundancy. However, they could not share protein substrates, at least for RIN4, PBL1, and BIK1 (Liu et al., 2021).

## Conclusion and perspective

The reversible nature represents one of the most important aspects of protein S-acylation. Since the discovery of the first S-acyltransferase, Akr1 from yeast in 2002, research on protein S-acylation has been accelerated at a remarkable speed in both mammals and plants (Roth et al., 2002; Li and Qi, 2017; Zheng et al., 2019; Hemsley, 2020). However, the advancement of S-acylation studies in plants still lags far behind compared to that in mammals in almost every aspect, including S-acylation proteomics, functional and regulation study of S-acylation-related enzymes, as well as their corresponding substrates. The knowledge from other organisms sheds light on the plant S-acylation research, although there are still many unanswered questions.

The cycle of S-acylation and de-S-acylation provides a molecular mechanism for membrane-associated proteins to undergo cycling and trafficking between different cell compartments. In Medicago, MfNACsa translocation is possibly mediated by de-S-acylation through MtAPT1 (Duan et al., 2017). In *Arabidopsis*, RIN4, PBS1, PBL1, HIR2, and BIK1 also undergo both S-acylation and de-S-acylation processes. The sensing factors in response to stimuli, regulatory mechanisms, and functions remain to be in-depth investigated for the dynamic change.

Interestingly, ABHD17s themselves are S-acylated in mammals, which is possibly catalyzed by PATs (Yokoi et al., 2016). Similarly, ABAPT proteins could be possibly S-acylated by plant PATs. Membrane association of ABHD17s facilitated by S-acylation enables ABHD17s to de-S-acylate other membrane proteins. In turn, the increased de-S-acylating activity of the membrane-anchored ABHD17s may reduce the auto-S-acylation of PAT proteins in the DHHC domain, which is essential for their PAT activities. It is possible that a feedback inhibition loop between PATs and ABAPTs may regulate the proteome-level S-acylation.

Compared to humans and yeast, research progress about plant PATs has been very limited. Other forms of PTMs, including phosphorylation, S-acylation, ubiquitination, acetylation, and methylation, could regulate the stability, localization, and function of several PAT proteins in mammals (Zmuda and Chamberlain, 2020). However, in plants, most of the PAT studies to date are limited to phenotypic observations and substrate validations. Precise regulatory mechanisms for PAT activities, substrate specificity, and responses to external stimuli are poorly understood, which are interesting and important questions needed to be answered in the future.

ABAPT protein family has 11 members, and their diverse subcellular localizations allow them to catalyze many substrates in different cell compartments. However, considering the large number of S-acylated proteins in the plant (Hemsley et al., 2013; Srivastava et al., 2016; Zhou et al., 2020; Kumar et al., 2022), it remains obscure how many proteins actually undergo palmitate cycling in plant cells and how their S-acylation status respond to stimuli. Based on the above knowledge, we could put forward the following hypotheses: (1) ABAPTs are not substrate-specific enzymes, each of which can de-S-acylate a wide range of substrates; (2) other acyl thioesterases, homologs to APTs, or a completely new acyl thioesterases family may exist in plant, which is waiting to be discovered; and (3) not all S-acylated proteins undergo de-S-acylation. Clearly, future efforts are needed to explore the substrate specificity, redundancy between ABAPT isoforms, and physiological roles of each ABAPT enzyme. The chemical tools, S-acylation process inhibitor 2-bromopalmitate and APT inhibitors Palmostatin B/M for instance, are able to overcome the redundancy of proteins and advance our understanding of the S-acylation function without gene mutations (Davda and Martin, 2014; Remsberg et al., 2021). At the same time, the specificity and selectivity of inhibitors against PATs, APTs, and ABAPTs need to be investigated.

The substrate specificity of both PATs and ABAPTs deserves more attention. Future work on S-acylation in plants should focus on determining how S-acylation and de-S-acylation impact substrate protein function. Revealing the determination and regulation mechanisms of the dynamics of S-acylation and the biological functions it impacted will help us better understand the significance of this lipid modification in plants.

## Author contributions

All authors listed have made a substantial, direct, and intellectual contribution to the work and approved it for publication.

## Funding

This paper and related research are funded by the Shuishan Scholars Program of Nanjing Forestry University (grant no. 163010299).

## Conflict of interest

The authors declare that the research was conducted in the absence of any commercial or financial relationships that could be construed as a potential conflict of interest.

## Publisher’s note

All claims expressed in this article are solely those of the authors and do not necessarily represent those of their affiliated organizations, or those of the publisher, the editors and the reviewers. Any product that may be evaluated in this article, or claim that may be made by its manufacturer, is not guaranteed or endorsed by the publisher.
